# Intra-and interobserver reliability of a modified distraction test based on digital images to assess lower eyelid horizontal tension

**DOI:** 10.5935/0004-2749.20200018

**Published:** 2020

**Authors:** Daniella de Paiva Almeida Stuchi, Juliana Rossato, Francisco José de Lima Bocaccio, Fernando Procianoy

**Affiliations:** 1 Departamento de Oftalmologia, Faculdade de Medicina, Pontifícia Universidade Católica, Campinas, SP, Brazil; 2 Hospital de Clínicas de Porto Alegre, Porto Alegre, RS, Brazil; 3 Departamento de Oftalmologia e Otorrinolaringologia, Faculdade de Medicina, Universidade Federal do Rio Grande do Sul, Porto Alegre, RS, Brazil

**Keywords:** Eyelids, Eyelid diseases, Ectropion, Entropion, Digital image, Pálpebras, Doenças palpebrais, Ectrópio, Entrópio, Imagem digital

## Abstract

**Purpose:**

Inferior eyelid laxity is classically evaluated using “snap-back” and “distraction” tests. This study aimed to assess the reproducibility of the technique used to indirectly quantify the horizontal tension in the lower eyelids using digital image processing.

**Methods:**

This longitudinal study was conducted to assess the reproducibility of a new technique that quantifies the horizontal tension in the lower eyelid. The study was conducted at the Hospital das Clínicas of Porto Alegre. The protocol was established by two trained ophthalmologist examiners, allowing intraand interobserver agreement analyses. Image acquisition was done in two stages: the first image was captured with the eyelid in primary gaze position and the second with the eyelid in traction position. All images and measurements were processed using Image J 1.33µ software from the National Institute of Health. The Bland-Altman method, intraclass correlation coefficients, concordance correlation coefficients, and technical measurement error were used to evaluate reproducibility.

**Results:**

The study participants comprised healthy individuals with no ophthalmologic pathologies. The measurements obtained in the neutral position showed a slightly higher agreement than those obtained in the traction position. The mean difference between the measurements performed in the traction position was 0.028 ± 0.7 mm and 0.014 ± 0.9 mm in the intraand interobserver analyses, respectively. The Bland-Altman method demonstrated adequate confidence limits for both measurements. Correlation coefficients for measurements varied between 0.87 [95% confidence interval (CI) 0.68-0.95] and 0.91 (95% CI 0.77-0.97) in the neutral position and between 0.72 (95% CI 0.37-0.89) and 0.76 (95% CI 0.4-0.91) in the traction position.

**Conclusion:**

A high intraand interobserver concordance was observed in the studied method to quantify lower eyelid tension. The proposed method is simple and easily reproducible, and to the best our knowledge, this is the first method that quantifies lower eyelid horizontal tension on the basis of digital image processing. This modified distraction test might be useful in studies quantifying lower eyelid horizontal tension.

## Abstract (pt)

**Objetivo:**

A frouxidão palpebral inferior é avaliada classicamente por meio de testes de “*snap-back*” e “*distraction test*”. O objetivo deste estudo foi avaliar a reprodutibilidade da técnica utilizada para quantificar indiretamente a tensão horizontal nas pálpebras inferiores através do processamento digital de imagens.

**Métodos:**

Este estudo longitudinal foi realizado para avaliar a reprodutibilidade de uma nova técnica que quantifica a tensão horizontal na pálpebra inferior. O estudo foi realizado no Hospital das Clínicas de Porto Alegre. O protocolo foi estabelecido por dois examinadores oftalmologistas treinados, permitindo análises de concordância intra e interavaliador. A aquisição de imagens foi feita em duas etapas: a primeira imagem foi capturada com a pálpebra na posição primária do olhar e a segunda com pálpebra tracionada. Todas as imagens e medições foram processadas usando o software Image J 1.33µ do National Institute of Health. O método de Bland-Altman, os coeficientes de correlação intraclasses, os coeficientes de correlação de concordância e o erro técnico da medida foram utilizados para avaliar a reprodutibilidade.

**Resultados:**

Os participantes do estudo foram indivíduos saudáveis e sem patologias oftalmológicas. As medidas obtidas na posição neutra mostraram concordância levemente maior do que as obtidas na posição tracionada. A diferença média entre as medidas realizadas na posição tracionada foi de 0,028 ± 0,7mm e 0,014 ± 0,9mm nas análises intra e interobservadores, respectivamente. O método de Bland-Altman demonstrou limites de confiança adequados para ambas as medidas. Os coeficientes de correlação para as medidas variaram entre 0,87 [intervalo de confiança de 95% (IC) 0,68-0,95) e 0,91 (IC 95% 0,77-0,97) na posição neutra e entre 0,72 (IC 95% 0,37-0,89) e 0,76 (IC 95% 0,46-0,91) na posição tracionada.

**Conclusão:**

Observou-se elevada concordância intra e interobservador no método estudado para quantificar a tensão palpebral inferior. O método proposto é simples e facilmente reproduzível, e, do melhor modo possível, este é o primeiro método que quantifica a tensão horizontal da pálpebra inferior com base no processamento digital de imagens. Este teste de distração modificado pode ser útil em estudos que quantifiquem a tensão horizontal da pálpebra inferior.

## INTRODUCTION

The lower eyelid is a dynamic structure suspended by a fibroligamentous sling that is supported by the medial and lateral canthal tendons, tarsus, lower lid retractors, and orbicularis oculi muscle. Horizontal eyelid tension is defined as the tension or pressure exercised on the ocular surface and cornea^(1)^.

Lower eyelid malposition frequently occurs in the elderly population, and horizontal eyelid laxity is one of the primary related causes. Such a condition may inflict not only unpleasant cosmetic consequences but also improper ocular exposition, resulting in related visual sequelae.

Enhanced comprehension of the lower eyelid anatomy and physiology is paramount in understanding the process that leads to eyelid laxity and in determining the adequate surgical technique for its correction.

The lower eyelid margin is normally positioned just above the lower limbus level. The lateral canthal tendon is normally positioned 2-4 mm above the medial canthal tendon, forming a lateral canthal angle of approximately 60°^(2)^.

The lower eyelid can be divided, from anterior to posterior, into the anterior, middle, and posterior lamellae. The anterior lamella comprises very thin skin tissue, pre senting no underlying subcutaneous fat opposing the orbicularis oculi muscle; the latter can be further divided into palpebral and orbital orbicularis muscles. The palpebral orbicularis muscle has been reported to play a significant role in the lower lid apposition to the globe and lacrimal pump^(3)^.

The degree of lower eyelid laxity can be clinically evaluated using “snap-back” and “distraction” tests. In the snap-back test, the lower eyelid is pulled down with the index finger to assess the speed of the eyelid’s return to its original position, and if blinking is needed to facilitate the repositioning. In the distraction test, the lower eyelid skin is delicately grasped and distracted from the eyeball with the index finger and thumb. The distraction is considered normal when the distance from the cornea is set at <2 mm; values >6 mm indicate ligament laxity.

### Pathogenesis of eyelid laxity

Although the palpebral position is affected by several factors, the most remarkable changes usually occur due to senile involution^(4)^. Pessa et al.^(5)^ have demonstrated that with aging, the lower lateral orbit increases in vertical dimension, whereas the vertical maxilla shortens, diminishing in projection, thus, causing the midface to shift inferiorly. This displacement may lead to a lateral bow of the eyelid, causing the lower portion to drift medially and inferiorly. With advancing age, lower eyelid laxity may be caused by the attenuation of the traction between the lateral canthus and its attachments to the lower eyelid. Bowing of the temporolateral aspect of the lower eyelid may divert it from the surface of the globe, which may result in ectropion and potentially lead to dry eye symptoms due to increased exposure of the globe.

Over time, several instruments have been developed to quantify the palpebral tension. Fu et al.^(1)^ have designed a device using a clamp placed on the eyelashes, two force transducers, an electronic interface, and a computer for data acquisition and control.

Fu et al.^(1)^ inserted a latex sensor into the patient’s lower fornix, transmitting the resulting force to a digital pressure gauge; using this method, they measured eyelid tension on the basis of the hydraulic conduction principle. The abovementioned devices are not only extremely unviable but also scantly replicated. The only attempt to objectively measure eyelid tension *in vivo* has been made by Vihlen et al.^(6)^ and Wilson et al.^(7)^; however, they experienced several shortcomings with the instrument used by them. By holding the eyelid and recording the force on the displacement curve, excessive movements of the head or eyes lead to a large number of unacceptable results. Furthermore, the fitting of straight lines through measurement charts was made visually and over varying displacement distances, resulting in inaccuracy. Martin^(8)^ has also reinforced that the method and instruments used by Vihlen et al.^(6)^ and Wilson et al.^(7)^ may have artificially augmented palpebral tension measurement.

Nowadays, methods utilizing digital images and computerized evaluations are increasingly being used because they are more reliable and allow details of aging^(9,10)^.

Thus, this study was conducted to assess and quantify eyelid tension in individuals without ocular alterations using digital image processing evaluated through a computer program.

## METHODS

This prospective, observational study, conducted from October to February 2017, comprised 16 individuals aged between 25 and 50 years who visited the special outpatient clinics of the Hospital de Clínicas de Porto Alegre (UFRGS).

Lower eyelid tension was evaluated by taking digital images of the face captured in the primary eye position (first photographic register) and after the observer exerted digital traction in the central region of the lower eyelid (second photographic register). These images were obtained using a *Sony Cyber-shot* DSC-WX70 digital camera, with individuals sitting with their chin and forehead properly propped up on a slit-lamp table. The camera was positioned on a suitable tripod in a fixed position 40 cm from the slit-lamp table, with all measurements performed in the same position and angle.

**Figure 1:**
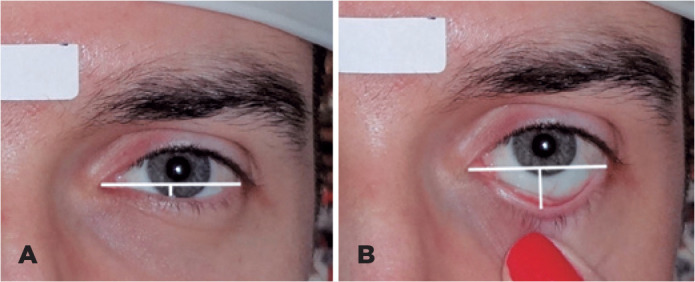
(A) Lower eyelid margin in a neutral position; (B) Traction of the lower eyelid.

Measurements: Photographic acquisition and measurements were performed by the same observer twice at distinct moments and then by a different observer at another moment. Each image was rotated and cropped to ensure that the baseline had zero angles, and a straight line was drawn starting from the lowest aspect of the medial canthus (baseline). Measurements were performed from the center of the baseline to the lower eyelid margin in a neutral position following digital traction of the lower eyelid (Figure 1). The difference between the two measures was considered as the amount of displacement in the lower eyelid that represented the eyelid horizontal tension. Measurements were processed using software Image J 1.33µ from the National Institute of Health.

The individuals who permitted the use of their captured images for study, did not have ocular abnormalities that could alter the palpebral position, and had never undergone ocular or palpebral surgeries were included in the study.

Initial measurements of the eyelid tension were described as mean values and standard error. For the interobserver agreement analysis, only the first measurement was considered.

Student’s *t*-test was used for paired measurements and, if significant, used to provide the first evidence of poor agreement between the measurements. The Bland-Altman method was used for the visual evaluation of the reliability of measurements and agreement limits^(11,12)^. To assess the magnitude of intraand interobserver agreement, intraclass correlation coefficients (ICCs), concordance correlation coefficients (CCCs), and technical mea surement error (TEM) as well as their respective 95% confidence intervals (CIs) were described^(13)^. ICC was chosen for full agreement and single measurements, with the two-way mixed model used for the intraobserver analysis and the one-way random model used for the interobserver analysis^(14)^. CCC was calculated using the method described by Lin^(15)^. Coefficients of >0.75 were considered good and satisfactory for the measured values. The absolute TEM was calculated based on the following equation:


$$\text{ Absolute }TEM=\sqrt{\sum d_{n}^{2}/2n}$$

Where: d= difference of measures; n= number of measures

Next, the relative TEM was calculated from the division between the absolute TEM and the arithmetic mean of the mean values of each pair for individual observations and expressed as a percentage^(16)^.

The analyses were performed using SPSS software (Statistical Package for Social Sciences) Version 20.0 and R studio (Integrated Development Environment for R) version 1.0.143. A two-tailed p-value of <0.05 was considered significant.

## RESULTS

Table 1 shows the mean values observed in the neutral and traction positions according to the two stages of the study. In general, significant differences between the average values observed in the two stages of the study could not be determined. The mean differences observed between the palpebral measurements in the neutral position were 0.028 ± 0.07 mm (paired *t*-test=0.37; p=0.72) and 0.143 ± 0.09 mm (paired *t*-test=0.15; p=0.88) for intraand interobserver analyses, respectively. The mean differences between the measurements in the traction position were slightly higher in the intraobserver analysis, being 0.101 ± 0.19 mm (paired *t*-test=-0.53; p=0.60), than those in the interobserver analysis, being 0.163 ± 0.15 mm (paired *t*-test=-0.06; p=0.31) (Table 1).

**Table 1:** Mean values and standard error of eyelid position measures

Eyelid	Observer 1	Observer I	Observer 1		Observer 11
**position**	**(1^st^ measure)**	**(2^nd^ measure) (Difference)**		**Observer 11**	(Difference)^*^
Neutral	1.76 ± 0.18	1.73 ± 0.18	0.028 ± 0.07	1.74 ± 0.18	0.014 ± 0.09
Tensioned	4.74 ± 0.25	4.84 ± 0.26	0.101 ± 0.19	4.91 ± 0.19	0.163 ± 0.15

* Difference from Observer I (1^st^ measure).

**Figure 2:**
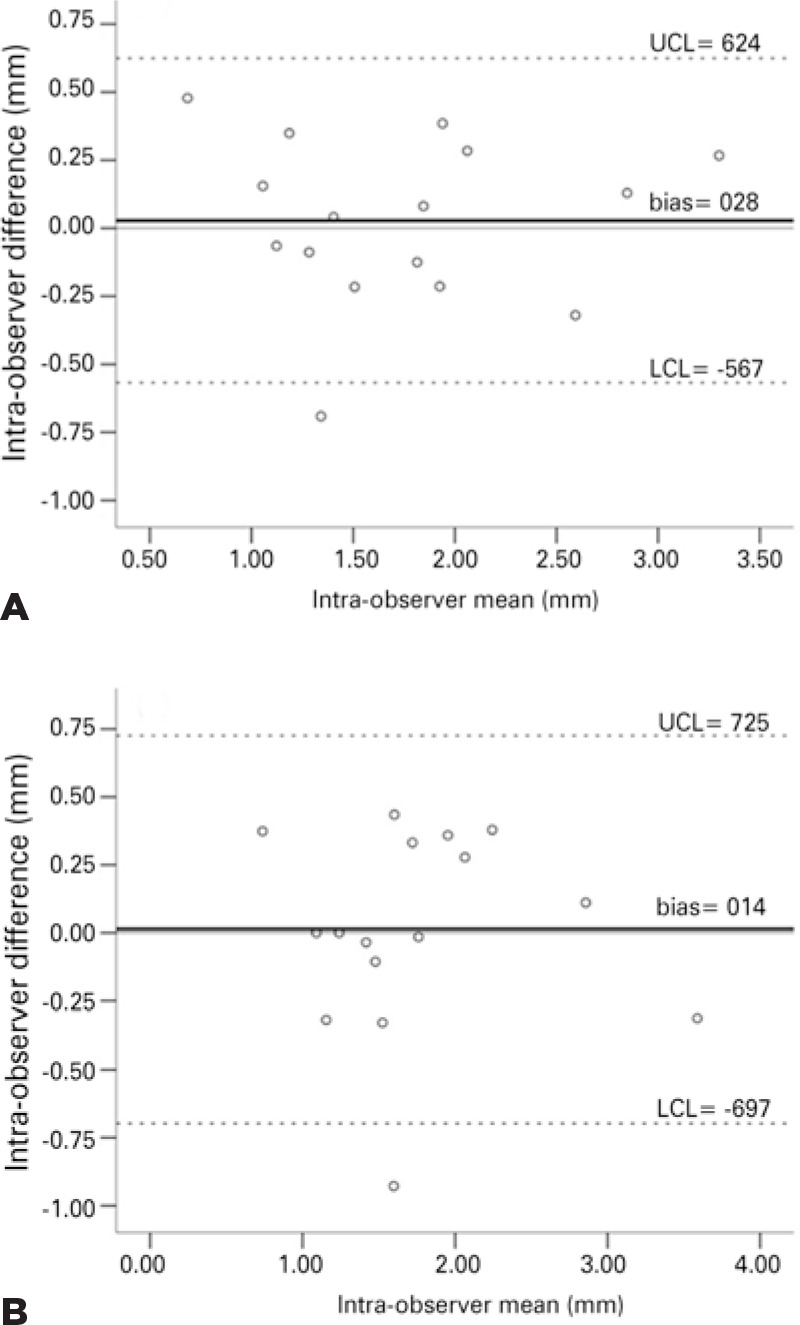
Bland-Altman^(12)^ plot of intra- (A) and interobserver (B) measurements obtained in the neutral position

Figure 2 presents an overview of intraand interobserver agreement of the eyelid measurements in the neutral position according to the Bland-Altman method. Observations occurring outside the obtained confidence limits were limited. Nevertheless, the presence of intraobserver (r=0.00; p=1.0) or interobserver (r=-0.3; p=0.91) “systematic error” was not observed, as evidenced by significant correlations between the individual difference (bias) and the measurement size (mean).

**Figure 3:**
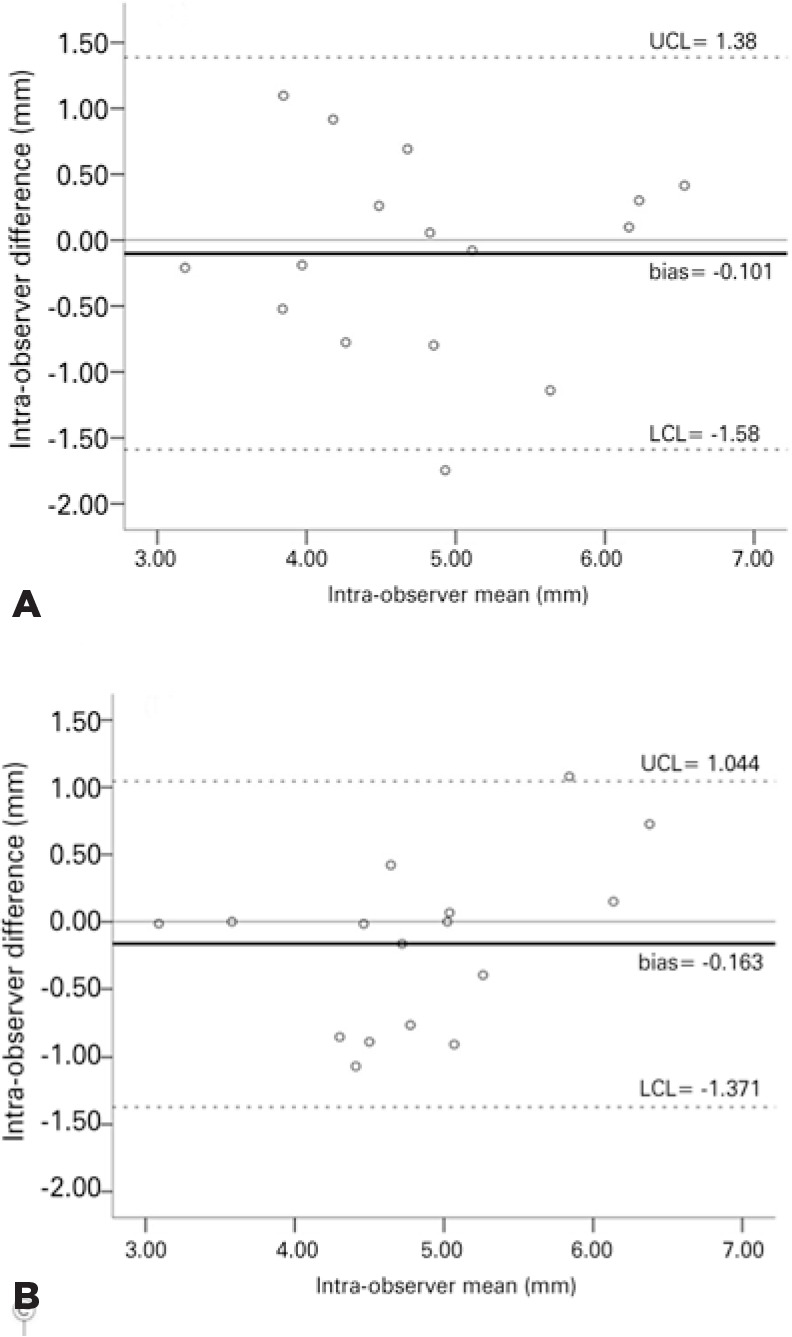
Bland-Altman^(12)^ plot of intra- (A) and interobserver (B) measurements obtained in the traction position

Figure 3 presents an overview of intraand interobserver agreement of the eyelid measurements in the traction position according to the Bland-Altman method. The findings obtained with the eyelid in the neutral position showed that only a few observations occurred outside the obtained confidence limits; therefore, the presence of intra- (r=-0.3; p=0.91) or interobserver (r=0.41; p=0.11) “systematic error” was not observed.

ICC and CCC were used for eyelid tension measurements in the neutral and traction positions. In general, measurements performed in the neutral position showed higher coefficients than those in the traction position. The correlation coefficients and agreement in the neutral position ranged from 0.873 to 0.915, indicating good or excellent reliability of the study participants (Table 2). TEM in the neutral position was 0.21 mm (11.9%) and 0.25 mm (14.2%) for the intraand in terobserver analyses, respectively.

**Table 2:** Intraclass correlation coefficient and concordance correlation coefficient of eyelid tension measures

	Neutral position		Pulled position	
	Index	95% CI	Index	95% CI
Intraobserver				
ICC	**0.915**	**0.77-0.97**	**0.735**	**0.39-0.90**
CCC	**0.909**	**0.76-0.97**	**0.722**	**0.37-0.89**
Interobserver				
ICC	**0.881**	**0.69-0.96**	**0.765**	**0.46-0.91**
CCC	**0.873**	**0.68-0.95**	**0.753**	**0.47-0.89**

CI= Confidence interval/ ICC= Intraclass correlation coefficient; CCC= Conc ordance correlation coefficient.

The coefficients observed in the traction position varied between 0.722 and 0.765, indicating good reliability of the study participants. Notably, the interobserver analysis showed slightly higher coefficients than the intraobserver analysis (Table 2). TEM in the traction position was 0.52 mm (10.9%) and 0.43 mm (9.1%) for the intrainterobserver analyses, respectively.

## DISCUSSION

In this study, we aimed to indirectly measure the lower eyelid tension using digital image processing. Ideally, this tension should be measured directly, but this is impractical due to the difficulty of acquiring an apparatus that performs the traction of the lower eyelids without causing patient discomfort. Digital image processing techniques have been successfully used to examine the palpebral contour in different pathologies of the eyelid position^(17)^. Thus, traction was performed with the examiner’s right thumb on the patient’s lower eyelid to evaluate the palpebral tension. A photograph was taken while performing the test, and the images were sent to a computer equipped with software for data editing and measurement (Image J 1.33µ).

The results of the data obtained by the examiners were evaluated considering the agreement and reliability of the test performed using the Bland-Altman method. The obtained data did not show a significant difference.

Palpebral tension has been known to decrease due to aging, pathologies, and drug use, among others. Therefore, the test in question may help identify the fundamental method in future studies.

The high intraand interobserver reliability found in this study was due to the ease of application of the technique and analysis of the results. Thus, the modified test to evaluate palpebral tension in the lower eyelid on the basis of digital images proved to be a reliable and feasible modality. The findings of this study can be used for future tests requiring such measurements, and to our knowledge, this is the first method that quantifies the lower eyelid horizontal tension on the basis of digital image processing.
